# Selection against Spurious Promoter Motifs Correlates with Translational Efficiency across Bacteria

**DOI:** 10.1371/journal.pone.0000745

**Published:** 2007-08-15

**Authors:** Jeffrey L. Froula, M. Pilar Francino

**Affiliations:** Evolutionary Genomics Program, DOE Joint Genome Institute, Walnut Creek, California, United States of America; Indiana University, United States of America

## Abstract

Because binding of RNAP to misplaced sites could compromise the efficiency of transcription, natural selection for the optimization of gene expression should regulate the distribution of DNA motifs capable of RNAP-binding across the genome. Here we analyze the distribution of the −10 promoter motifs that bind the σ^70^ subunit of RNAP in 42 bacterial genomes. We show that selection on these motifs operates across the genome, maintaining an over-representation of −10 motifs in regulatory sequences while eliminating them from the nonfunctional and, in most cases, from the protein coding regions. In some genomes, however, −10 sites are over-represented in the coding sequences; these sites could induce pauses effecting regulatory roles throughout the length of a transcriptional unit. For nonfunctional sequences, the extent of motif under-representation varies across genomes in a manner that broadly correlates with the number of tRNA genes, a good indicator of translational speed and growth rate. This suggests that minimizing the time invested in gene transcription is an important selective pressure against spurious binding. However, selection against spurious binding is detectable in the reduced genomes of host-restricted bacteria that grow at slow rates, indicating that components of efficiency other than speed may also be important. Minimizing the number of RNAP molecules per cell required for transcription, and the corresponding energetic expense, may be most relevant in slow growers. These results indicate that genome-level properties affecting the efficiency of transcription and translation can respond in an integrated manner to optimize gene expression. The detection of selection against promoter motifs in nonfunctional regions also confirms previous results indicating that no sequence may evolve free of selective constraints, at least in the relatively small and unstructured genomes of bacteria.

## Introduction

Increased efficiency of cellular functions may impose selective pressures on many factors beyond the amino acid sequences of the cell's proteome. The capacity to produce the necessary proteins at the right time and in the required amount is a system-wide cellular property that requires integration of numerous signals and processes. In bacteria, finely tuned gene expression is the main recourse for immediate interaction with the environment, and should be selected to proceed in a rapid, precise and cost-efficient manner. The efficiency of bacterial translation is known to be under measurable selective pressure; this is evidenced by the fact that, in many bacteria, the most highly expressed genes utilize restricted sets of codons that allow for fast and accurate translation of mRNA, by virtue of their optimal interaction with the most abundant tRNA species for a given amino acid [1]–[5]. In addition, recent comparative analyses across numerous bacterial genomes have revealed that the abundance and variety of the tRNA gene sets encoded in a genome is highly correlated with the maximal growth rate of the bacterial species. The genomes of fast-growing bacteria contain large numbers of tRNA genes, with the diversity of the encoded tRNA set being low and well adjusted to the codon usage in that species. As a result, the codon usage bias in the highly expressed genes of fast growers is extreme [5].

The selective pressures operating on the general efficiency of transcription are less well understood. Although recent experimental studies suggest that the efficiency of transcription may be regulated at every stage of the process [6], [7], the frequency of transcription initiation is likely to remain the strongest determinant of total transcriptional output, as evidenced by the complexity of regulatory mechanisms operating at this step. In particular, rapid and accurate localization and binding of correct transcription promoter sites by the RNA polymerase (RNAP) holoenzyme may be one of the critical steps limiting the overall efficiency of gene expression, especially in bacteria, where genomic DNA is not protected by nucleosome-like structures. Spurious binding could result in draining of RNAP molecules away from real promoters, in wasteful attempts to initiate transcription at unproductive sites, or in the generation of unwanted transcripts [8]. However, promoter recognition is likely to be compromised by the fact that RNAP is capable of binding, and possibly initiating transcription, at a multitude of DNA sequences.

Although promoters follow general rules in terms of their nucleotide sequence, structure, length and position, many variations on the canonical promoter model allow for function. In the case of σ^70^ promoters, which regulate the majority of *E. coli* genes, the canonical model is characterized by two hexamers, centered around positions −35 and −10 from the +1 transcription initiation site and separated by 15 to 21 bp, which are bound by distinct regions of the σ^70^ molecule. Comparisons of numerous promoter sequences have defined TTGACA and TATAAT as the consensus sequences for the −35 and −10 promoter motifs [9], [10]. However, transcription initiation does not proceed from promoters having strict consensus sequences, because the binding of the RNAP to a perfect consensus promoter is so strong that it prevents the necessary steps towards promoter clearance and elongation initiation [11]. Rather, variations of the consensi are used which have lower but more functional binding strengths. On average, *E. coli* promoters preserve only 8 of the 12 canonical bases of the −35 and −10 hexamers [10], [12].

Given the variety of sites recognized by RNAP, promoter-like motifs are likely to appear across DNA sequences by neutral mutation and random genetic drift. Computer simulations have shown that this is indeed the case for eukaryotic transcription factor binding sites, which can appear neutrally within microevolutionary timescales [13]. Selection for efficient gene expression should therefore act to maintain low frequencies of potential RNAP-binding sites in locations where they are not needed. Several lines of evidence have emerged that suggest that selection may indeed operate to control the genomic distribution of DNA motifs capable of binding to σ^70^. In a series of recent computational analyses, it has been established that the densities of promoter-like sequence motifs differ between regulatory and nonregulatory regions of the genome for a majority of bacteria. The density of promoter-like sequences is high within regulatory regions, in contrast to coding regions and regions located between convergently-transcribed genes, where functional promoters are not required [14]–[16]. However, comparisons among genomic regions can't determine whether σ^70^ binding sites are over-represented in regulatory regions and/or under-represented in nonregulatory regions beyond their expected values. To address this issue, actual motif counts need to be compared to the expected occurrences of the motifs given the base composition of each type of region. Hahn, Stajich and Wray [8] conducted such an analysis for the consensus promoter sequences TTGACA and TATAAT, showing that the numbers of these words are below the expectations in both coding and noncoding regions of numerous bacterial genomes. This implies that natural selection acts to remove spurious occurrences of the consensus σ^70^-binding motifs.

Given that σ^70^ recognizes many sequences beyond its canonical consensi, we were interested in determining the collective behavior of motifs capable of σ^70^-binding, and in understanding the role of natural selection in shaping their genomic distribution. In order to investigate the specific selective pressures acting on the distribution of these motifs, we decided to study and compare their densities in different genomic regions for bacteria having different lifestyles, growth rates and levels of optimization of the translational process. Such a broad comparison is warranted because several factors indicate that all bacteria contain promoter motifs similar to those found in *E. coli,* particularly for the −10 region. RNAP is evolutionarily conserved across bacteria, and, in particular, the orthologues of σ^70^ can be clearly aligned and contain highly similar motifs for the recognition and binding of promoter sequences [17]. Recent computational analyses have shown that when genomes of bacterial species belonging to different phyla are searched with *E. coli* position frequency matrices, collections of promoter-like motifs are detected which are similar to those of *E. coli* in several respects, including consensus sequence and average score [15], [16]. Experimental evidence from several bacteria belonging to different phyla also indicates that the −10 box is conserved and displays the same consensus, although the −35 region may be more variable and can't be defined for all genes [18]–[21]. Accordingly, the −10 motif has been shown to be the most essential component of the promoter in *E. coli*, whereas the −35 motif can be replaced by other combinations of elements upstream of the −10 [22]–[24]. Therefore, we focused our analyses on the −10 motifs present in the genomes of numerous bacterial species belonging to distant phylogenetic groups.

## Results

### Over- and under-representation of −10 motifs in different genomic regions of *E. coli*

We first analyzed the distribution of −10 motifs capable of σ^70^-binding in the genome of *E. coli*. To this aim, we defined a list of 185 unique −10 hexamers (Table S1) derived from 584 experimentally detected σ^70^ *E. coli* promoters reported in RegulonDB [25]. We obtained the total number of occurrences of these hexamers for three distinct categories of genomic sequence: regulatory, coding and nonfunctional. In order to establish whether these sites are under or overrepresented in each sequence category, we compared the observed counts to the expected occurrences of the hexamers given the base composition of each type of region (see Methods).

**Figure 1:**
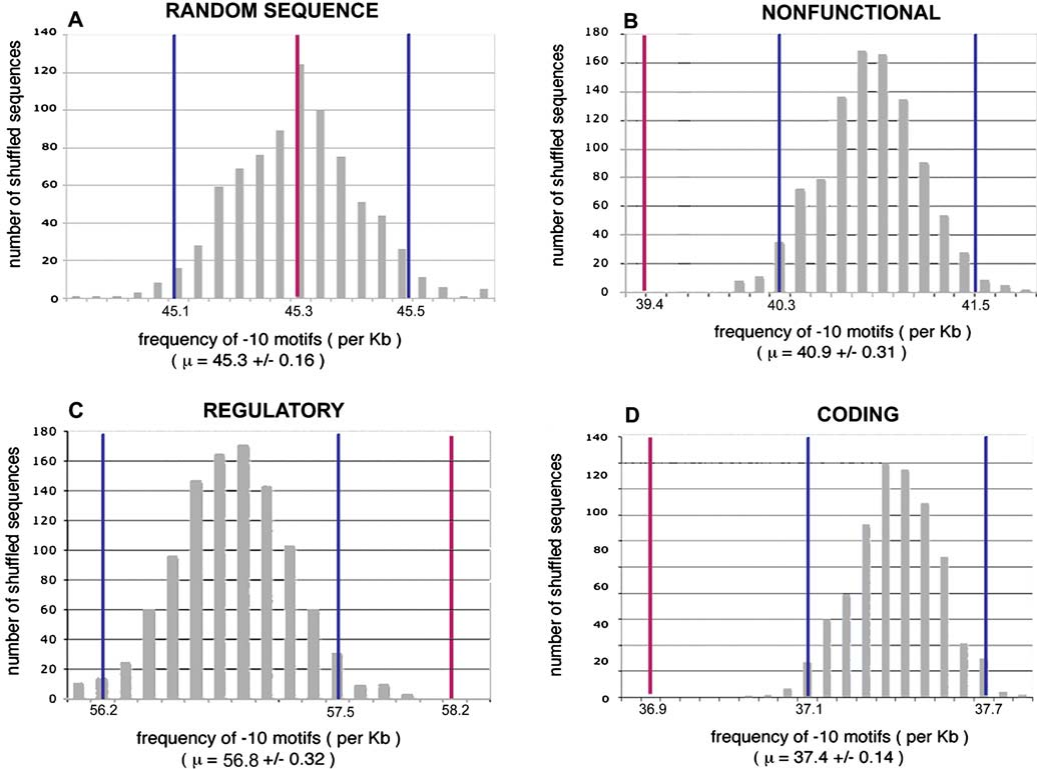
Comparison of observed and expected −10 motif frequencies in different genomic regions of *E. coli*. Distributions were obtained by counting and summing the occurrences of 185 −10 hexamers in sets of 1000 shuffled sequences. This procedure was performed independently for (A) randomly generated, (B) nonfunctional, (C) regulatory, and (D) coding sequences. In each panel, the blue lines represent ± 2 Standard Deviations (SD) from the mean value in the population of shuffled sequences and the red line represents the observed count in the unshuffled *E. coli* sequence.

Figure 1 compares the observed hexamer counts in *E. coli* with the values obtained for sets of 1000 shuffled sequences for each sequence category, as well as for a control set of randomly generated DNA sequence. The control shows that there were no procedural biases, as the observed value from the original random sequence lies within the curve for shuffled random DNA. The regulatory and nonregulatory sequences behave in an opposite manner. In regulatory regions, the observed total hexamer count is beyond any value obtained in the population of shuffled sequences, whereas the observed count in coding and nonfunctional DNA is below any of the simulated values. We therefore conclude that the hexamers corresponding to the −10 σ^70^-binding motif are significantly over-represented in regulatory regions and under-represented in nonregulatory regions.

This implies that, in *E.coli*, natural selection is favoring a high density of σ^70^-binding sites in regulatory regions, while keeping their frequencies low in other types of sequences. The under-representation of potential σ^70^-binding sites in nonregulatory regions is likely to be related to selection against spurious RNAP-binding to achieve higher transcriptional efficiency. The functional and evolutionary causes that might be responsible for maintaining high densities of promoter-like signals in regulatory regions have been previously described [14]–[16]. Some of the σ^70^-binding sites in regulatory regions could be part of distinct functional promoters that direct transcriptional initiation under different environmental conditions. Alternatively, these sites may not serve as locations of transcription initiation, but rather may affect the function of the real start site in different ways. For instance, these sites could bind RNAP to maintain a local abundance of RNAP molecules close to the transcription start site. They could also play a more specific regulatory role, by affecting the interactions of the functional promoter with regulatory proteins, or by causing the transcription complex to pause after initiation during the early steps of elongation. Finally, several modes of natural selection could operate over regulatory regions that would result in a rapid turnover of functional binding sites and maintenance of numerous cryptic signals, such as compensatory selection, stabilizing selection or selection for robustness to mutation.

**Figure 2:**
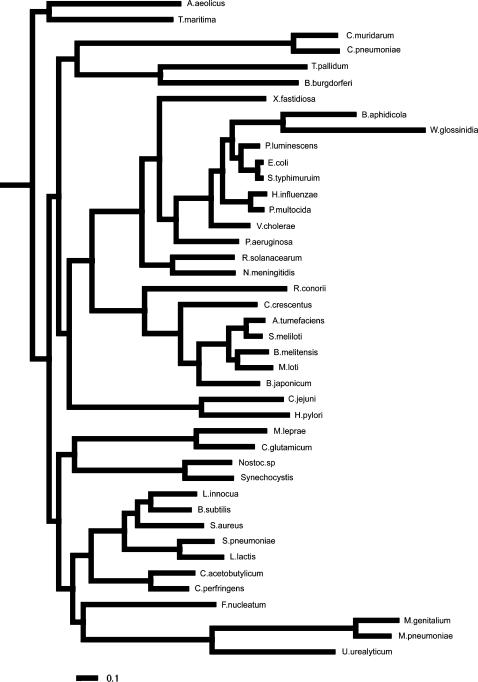
Phylogeny of the 42 bacterial species analyzed. Phylogeny based on a set of 51 genes present in single copy in most bacterial genomes. Concatenated multiple sequence alignments were used as input for the bayesian tree reconstruction program MrBayes [50]. The tree topology and branch lengths shown correspond to the most likely tree obtained in two independent runs of MrBayes spanning 250,000 generations each. All clades were recovered with 100% posterior probability. The tree is rooted on the branch leading to the hyperthermophiles *Aquifex aeolicus* and *Thermotoga maritima*.

In order to achieve some understanding of the relative importance of selection for and against potential σ^70^-binding sites in different bacteria, we have analyzed the distribution of −10 σ^70^ motifs in different regions of 41 additional bacterial genomes (Table 1 and Fig. 2).

**Table 1:** Bacterial species analyzed and their general features.

Species	Accession	tRNA Genes	Growth Rate	Genome Size (Mb)
Ureaplasma urealyticum	NC_002162	30	I	0.75
*Buchnera aphidicola*	NC_004545	32	S	0.62
*Borrelia burgdorferi*	NC_001318	33	S	1.52
Rickettsia conorii	NC_003103	33	S	1.27
*Wigglesworthia glossinidia*	NC_004344	34	S	0.7
*Helicobacter pylori*	NC_000915	36	I	1.67
*Mycoplasma genitalium*	NC_000908	36	S	0.58
*Chlamydia muridarum*	NC_002620	37	S	1.08
*Mycoplasma pneumoniae*	NC_000912	37	S	0.82
*Chlamydophila pneumoniae*	NC_002179	38	S	1.23
*Aquifex aeolicus*	NC_000918	44	S	1.59
*Campylobacter jejuni*	NC_002163	44	I	1.64
*Synechocystis*	NC_000911	44	S	3.95
*Mycobacterium leprae*	NC_002677	45	S	3.27
*Treponema pallidum*	NC_000919	45	S	1.14
*Thermotoga maritima*	NC_000853	46	I	1.86
*Fusobacterium nucleatum*	NC_003454	47	S	2.17
*Xylella fastidiosa*	NC_002488	50	S	2.73
*Bradyrhizobium japonicum*	NC_004463	51	S	9.11
*Caulobacter crescentus*	NC_002696	51	I	4.02
*Agrobacterium tumefaciens*	NC_003062	53	I	5.67
*Brucella melitensis*	NC_003317	54	I	3.29
*Mesorhizobium loti*	NC_002678	54	I	7.6
*Sinorhizobium meliloti*	NC_003047	56	I	6.69
*Haemophilus influenzae*	NC_000907	57	F	1.83
*Pasteurella multocida*	NC_002663	57	I	2.26
*Ralstonia solanacearum*	NC_003295	57	S	5.81
*Streptococcus pneumoniae*	NC_003098	58	F	2.04
*Neisseria meningitidis*	NC_003112	59	I	2.27
*Corynebacterium glutamicum*	NC_003450	60	?	3.31
*Staphylococcus aureus*	NC_002758	60	F	2.9
*Lactobacillus lactis*	NC_002662	61	I	2.37
*Pseudomonas aeruginosa*	NC_002516	64	I	6.26
*Listeria innocua*	NC_003212	66	F	3.09
*Clostridium acetobutylicum*	NC_003030	73	F	4.13
*Photorhabdus luminescens*	NC_005126	85	F	5.69
*Bacillus subtilis*	NC_000964	86	F	4.21
*Salmonella typhimurium* LT2	NC_003197	86	F	4.95
*Escherichia coli* K12	NC_000913	86	F	4.64
*Nostoc sp.*	NC_003272	89	S	7.21
*Clostridium perfringens*	NC_003366	96	F	3.09
*Vibrio cholerae*	NC_002505	98	F	4.03

Growth rates correspond to optimal generation times as follows: slow (S)>3 h; intermediate 3 h≤(I)≥40 min, and fast (F)<40 min. Estimates of the optimal generation times were obtained from [5].

### Over- and under-representation of −10 σ^70^ motifs in different bacterial genomes

Given that the motif responsible for binding the −10 promoter region is highly conserved in all bacterial σ^70^ orthologues, we used the collection of 185 −10 hexamers defined in *E. coli* for analyses of all bacterial species. For each genome, we defined regulatory, coding and nonfunctional regions as previously done for *E. coli* and we obtained the sum of the number of occurrences of each hexamer in every region (Tables S2, S3 and S4). Shuffled sequences were generated for every sequence category in every genome analyzed, in order to produce independent genome-specific distributions of expected hexamer counts.

**Figure 3:**
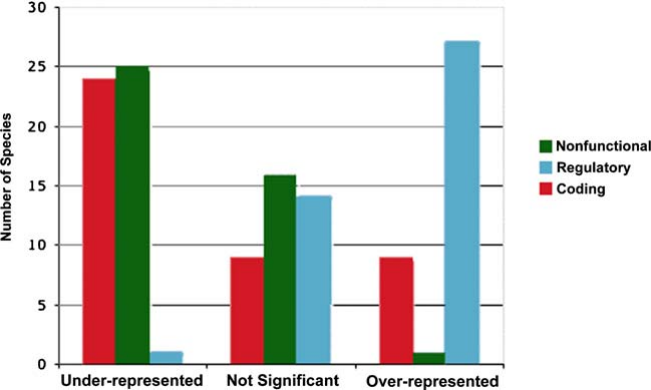
Patterns of over- and under-representation of −10 motifs across species. For every species, observed and expected −10 motif frequencies in the three different genomic regions were compared as in Figure 1. Over- and under-representation were considered significant when the observed frequency was beyond ±2 SD from the mean value for shuffled sequences.

**Figure 4:**
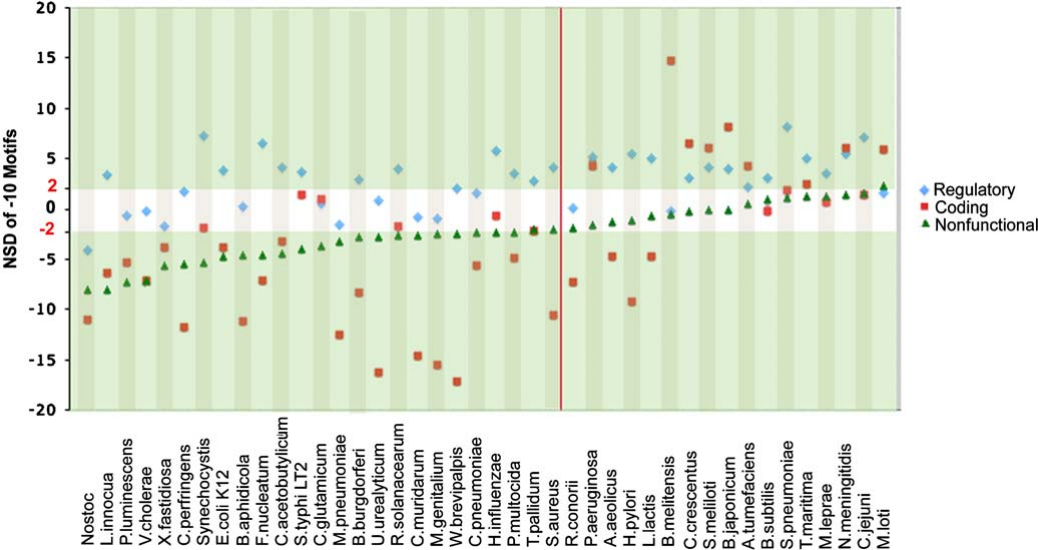
Deviations in the observed counts of −10 motifs in different genomic regions. NSD values measure the deviation in the observed counts of −10 motifs in numbers of standard deviations from the expected mean in the corresponding genome, and were obtained by comparing observed counts to distributions of motif frequencies in shuffled sequences. Species are ordered according to ascending NSD values for the nonfunctional regions. NSD values in the white band are not significantly different from expectation. The red line separates species with under-representation of −10 sites in nonfunctional regions (left) from those without (right).

Figure 3 shows the overall pattern obtained across bacterial genomes. Regulatory regions present a significant over-representation of −10 σ^70^ motifs in a majority of bacteria, whereas the same sites are significantly under-represented in a majority of the nonfunctional and coding regions. Figure 4 shows the deviations in the observed counts of −10 σ^70^ motifs measured in numbers of standard deviations from the expected mean (NSD), for the three types of genomic regions (see also Tables S2, S3 and S4). Species are ordered according to ascending NSD values for the nonfunctional regions. Because there is no reason to expect any selection for −10 σ^70^ motifs in nonfunctional regions, these NSD values may be interpreted as the baseline strength of selection against spurious binding in a given genome. Overall, the NSD in the three regions covary across genomes (regulatory vs. nonfunctional: r = 0.44 p<0.005; coding vs. nonfunctional: r = 0.52 p<0.001 and coding vs. regulatory r = 0.38 p<0.02). Moreover, in nearly all cases, the nonfunctional regions and the regulatory regions present distinguishable patterns of abundance of −10 sites. In the left area of the graph in Figure 4, genomes show the hallmark of selection against spurious sites (under-representation) in nonfunctional regions, whereas regulatory regions do not deviate from expectation or display an over-representation of −10 motifs. In contrast, in the right area of the graph, nonfunctional regions do not deviate from expectation, but most genomes display selection for −10 site over-representation in the regulatory regions. Thus, as a result of selection for −10 sites in the regulatory regions, selection against them in the nonfunctional regions, or the combination of both processes, the localization of RNAP molecules should be biased towards regulatory regions in all genomes. This indicates that the optimization of transcriptional efficiency via the distribution of potential −10 binding sites is an important selective constraint across bacteria. Remarkably, although under-representation of potential σ^70^ binding sites is the most common pattern in coding regions (24/42), there also exist several genomes with significant over-representations (9/42), indicating selection of these sites and suggesting some functional effect on the transcription process.

### Selection against spurious σ70 sites in nonfunctional regions correlates with translational efficiency

Next, we explored possible factors influencing the observed patterns of distribution of −10 σ^70^ sites in different genomes. The 42 bacterial species analyzed belong to many different phyla and have substantially different lifestyles reflected in their overall cellular and genomic features. In particular, the species considered show a large range of variation in growth rate as well as in the level of optimization of the translational process, as evidenced in the number of encoded tRNA genes (Table 1) and the degree of adaptive bias in codon usage [5].

Given that the bacterial cell is not highly compartmentalized, the two components of gene expression, transcription and translation, are tightly coupled, with ribosomes actively synthesizing polypetides from the 5′ ends of mRNAs while the 3′ ends have yet to be completed [26], [27]. The speed of gene expression should therefore depend on the speeds of both transcription and translation, and conditions, such as high growth rate, that select for an increased rate of gene expression should affect each of the two processes. Consequently, if σ^70^-binding site avoidance in nonregulatory regions is selected for in order to increase the speed of transcription, the degree of under-representation of these sites in a genome should respond to selection for high growth rate and vary in a manner that correlates with translational optimization.

The number of tRNA genes encoded in a bacterial genome is a good measure of the degree of optimization for translational speed. Given that the rate-limiting step during protein synthesis is the diffusion of charged tRNAs to the A site of the ribosome, where peptide bond formation is accomplished [28], a high concentration of tRNA molecules in the cell will allow for translation to occur more rapidly. Indeed, tRNA abundance varies widely across bacteria and has been shown to evolve rapidly and correlate strongly with maximal growth rate and adaptive codon usage [5], [29]. This indicates that this parameter effectively tracks the needs of bacteria for translational speed and efficiency.

**Figure 5:**
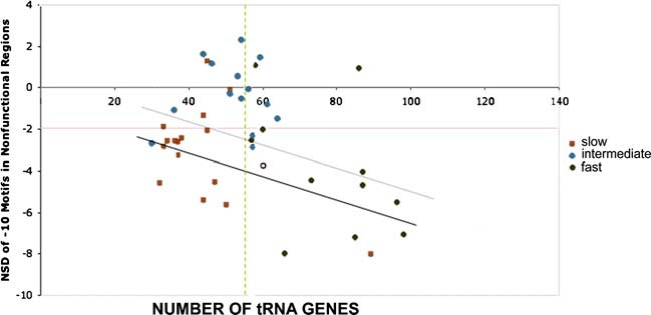
NSD of −10 motif counts in nonfunctional DNA correlate negatively with the number of tRNA genes in the genome. NSD values are defined as in the text and Figure 4. The red line represents −2 SD from the mean expected value of −10 motif counts in the nonfunctional DNA of the corresponding genome. The green line indicates the average number of tRNA genes in the genomes analyzed (55). The color-coding distinguishes species according to their maximal growth rate (see Table 1); no growth rate estimate was available for *Corynebacterium glutamicum* (not colored). Black dotted line: regression including all analyzed genomes (r = 0.38 and p<0.02). Black solid line: regression for the subset of genomes with NSD<−2 in nonfunctional DNA (r = 0.63 and p<0.001).

The abundance of tRNA genes varies greatly across the bacterial genomes employed in this analysis, ranging from 30 in *Ureaplasma urealyticum* to 98 in *Vibrio cholerae*, with an overall average of 55 tRNAs per genome (Table 1 and Fig. 5). The color-coding in Figure 5 distinguishes species according to their maximal growth rate to stress the association between this trait and the number of tRNA genes. Most of the slowest and fastest growers display binding site under-representation in nonfunctional regions (NSD<−2, below the red line) and are distributed across the range of tRNA values in accordance with their varying growth rates [5]. There is a distinct cluster at the extreme of low tRNA abundance (<40) containing the smallest genomes (≤1.5 Mb), which correspond to species that have independently evolved by genome reduction in several bacterial phyla and generally have very slow growth rates [30]. In contrast, most of the intermediate growers, with minimal generation times between one and three hours [5], present a clustered distribution deviating moderately from the average tRNA gene number but display no site under-representation in nonfunctional regions (NSD>−2, above the red line).

Even though σ^70^ site under-representation is more frequently detected in slow growers than in intermediate growers, when all species analyzed are considered the degree of deviation in nonfunctional DNA correlates negatively with the number of tRNA genes in the genome (r = 0.38 and p<0.02, dotted line in Fig. 5). This is consistent with the notion that both characters are affected by selection for high rates of gene expression, although the relative placement of slow and intermediate growers indicates that this selective pressure is not the only factor at play. As selection against σ^70^ sites in the nonfunctional DNA appears to be mostly absent from species with intermediate growth rates, we also analyzed separately the subset of slow and fast growers where this selection is actually detected (NSD<−2, below the red line). When only such genomes are included, the correlation between the degree of deviation in nonfunctional DNA and the number of tRNA genes increases (r = 0.63 and p<0.001, solid line in Fig. 5). In order to ensure that the obtained correlations are not due to phylogenetic relatedness, we employed a method that takes into account the topology and branch lengths of the phylogenetic tree relating the analyzed species (Fig. 2). This methodology implements a generalized least squares (GLS) model and likelihood ratio tests to resolve whether correlation between two characters is dependent on the underlying phylogeny [31], [32]. The correlation among NSD and tRNA genes is recovered when phylogeny is considered (p<0.05 for all species and p<0.0005 for those with NSD<−2).

## Discussion

Overall, the results of our analyses indicate that selection operates on almost all bacterial genomes to shape patterns of distribution of σ^70^-binding sites that distinguish regulatory and nonfunctional DNA. The distinct behavior of these regions may be due to three different situations: 1) selection for under-representation in nonfunctional regions, 2) selection for over-representation in regulatory regions, or 3) the joint effects of both processes. Selection for under-representation of σ^70^ sites is also detected in the coding regions of 24 of the 42 genomes analyzed. However, in contrast to the general trend, the coding regions of some genomes (9/42) do not avoid potential σ^70^ binding sites, but rather display an over-representation similar to that seen in regulatory DNA. This indicates that some form of natural selection is favoring the presence of σ^70^ sites in coding regions beyond random expectations. Although it is impossible to rule out that some selective pressure at the protein level may influence the occurrence of given nucleotide motifs in coding DNA, the fact that we took into account trinucleotide frequencies in generating our baseline expectations implies that only biases at the level of amino acid pairs or triplets could possibly influence our site counts. Therefore, it seems probable that selection for some other function, presumably related to transcription, is favoring the observed over-representation of −10 σ^70^ sites in coding DNA.

Of the possible explanations for σ^70^ site over-representation advanced for regulatory regions [14]–[16], only one seems to be equally applicable to coding DNA. It is very unlikely that σ^70^-binding sites in coding regions could function as promoters or regulate transcription by interaction with the transcription start site. However, these sites could affect the rate of transcription elongation by causing the elongation complex to pause at different points as it advances through a transcriptional unit, as has been demonstrated to occur in the earliest phase of elongation after promoter clearance [33], [34]. Recent experimental evidence indeed suggests that motifs with affinity for σ^70^ may influence the activity of the transcription elongation complex along the extent of a transcriptional unit [6], [7] . This could have a fine-tuning regulatory role, by modulating the speed of elongation and/or by providing pausing times at specific sites to allow for interaction with additional regulatory molecules.

The different types of selective pressures that can affect the presence of σ^70^ binding sites across the genome seem to be operating at different extents in different bacteria. Moreover, these patterns of selection are not independent of those affecting gene expression at the translational level, and their relationships to different bacterial lifestyles suggest that they may actually correspond to distinct biological needs. We will now summarize the patterns of selection on transcriptional and translational efficiency in different groups of bacteria and how they might relate to the general biology of the species in which they're found.

Selection against spurious binding is expected to be strongest in species that grow at the fastest rates and therefore require the highest speed and efficiency during transcription and translation. The genomes of fast-growing species bear the expected hallmarks of selective pressure for fast growth at the translational level, such as elevated numbers of tRNA genes (average 78; Table 1 and Fig. 5) and high adaptive biases in codon usage [5]. Here we show that 9 of the 11 analyzed genomes from species with minimum generation times under 40 minutes undergo selection against spurious sites in the nonfunctional DNA, to a degree which correlates well with their abundance of tRNA genes (Figs. 4 and 5). In fact, most fast-growing species respond to selection on −10 sites across all genomic regions, as these sites are also under-represented in the coding DNA and over-represented in the regulatory regions. Thus, selection against spurious binding is pervasive across the non-regulatory DNA of these genomes, but is often overturned in regulatory DNA, indicating that an excess of −10 sites at these regions can confer some advantageous function(s) related to gene expression even at high growth rate. However, it is interesting that the regulatory regions of the two fastest growers, with minimum generation times of 12 minutes -*Vibrio cholerae* and *Clostridium perfringens*- [5], do not deviate from the expected abundances of −10 sites, suggesting that the potential advantages conferred by over-representation of these sites are lost at the extreme of rapid growth.

Most of the analyzed genomes having minimum generation times between 40 minutes and 3 hours do not display any evidence of selection against spurious binding, suggesting that high efficiency during transcription is not critical at intermediate growth rates. Also absent from these genomes are the hallmarks of selection for efficiency at the translational level, as these species have moderate numbers of tRNA genes (average 52) and a moderate degree of adaptive bias in codon usage [5]. These observations suggest that selection on the general efficiency of gene expression is relaxed in species growing at intermediate rates. In contrast, most of these genomes show an over-representation of −10 sites in both the regulatory DNA and the coding regions (Tables S2, S3, S4 and Fig. 4). This suggests that bacteria growing at moderate speed do benefit from the potential advantages conferred by an excess of such sites at these regions, presumably through functional effects related to gene expression. In particular, these are the only species where over-representations of −10 sites are detected in the coding regions, suggesting that regulation of transcription elongation by σ^70^-induced pausing might be most effective at moderate rates of growth. The presence of −10 sites in coding regions could potentially diminish the efficiency of transcription initiation through competition with RNAP-binding sites in the upstream regulatory regions, which could be disadvantageous at very fast growth rates. But even the strongest over-representation of −10 sites in the coding regions can be compatible with a moderate rate of growth, as *Brucella melitensis*, for instance, has a minimal generation time of 2 h [5] in spite of exceeding its expected value of −10 sites in coding regions by nearly 15 NSD. In this context, it is important to notice that −10 sites may strongly exceed their expected value in the coding regions of a genome and still occur in numbers substantially lower than those detected in the regulatory DNA. This is due to the fact that GC content is higher in coding regions, which results in lower baseline expectations for the AT-rich −10 sites.

The slowest growers among the species analyzed are bacteria that can only survive in close association with a eukaryotic host. Most are animal symbionts or parasites whose lifestyle is completely host-restricted. As a consequence, these bacteria exist in small and subdivided populations prone to genetic drift, and are known to be subject to a process of genome reduction and degradation, commonly undergoing rapid sequence evolution with an AT biased mutational pressure [30], [35]. As expected, these reduced genomes (≤1.5 Mb; Table 1) bear no evidence of selection on translational efficiency, as they have low tRNA gene numbers (average 36) and display no adaptive biases in codon usage [5], [36], [37]. But, remarkably, these host-restricted species display significant under-representations of −10 sites in the nonregulatory regions of the genome, both coding and nonfunctional. This suggests that minimizing spurious binding is advantageous in these species even if there is no selective pressure for rapid growth. An alternative selective pressure for increasing efficiency during transcription may be energetic economy. Given that genome degradation results in general problems of protein stability and functionality [35], [38], [39], host-restricted species may be under strong selective pressure to perform basic cellular functions with limited numbers of active molecules. The avoidance of spurious binding could represent considerable energetic savings by allowing transcription to proceed with minimal numbers of RNAP molecules per cell. In contrast to nonregulatory regions, the abundances of −10 sites in the regulatory regions of most reduced genomes conform to expectation, with moderate deviations only in *Wigglesworthia glossinidia* (2.09 NSD) and the spirochaetes *Treponema pallidum* (2.78 NSD) and *Borrelia burgdorferii* (2.89 NSD). This suggests that selective pressures for site over-representation are of little effect in host-restricted bacteria. Recent evidence indicates that several other aspects of gene regulation are partially degraded in reduced genomes. Losses of certain promoter sequences, specialized σ factors and regulatory proteins have been documented [39]–[41]. In addition, the collections of predicted −10 and −35 promoter motifs in these genomes deviate from those of most other bacteria, in that they have lower average scores and G to T changes in the consensus sequences [15], [16]. Such a pattern of moderate degradation of regulatory functions in host-restricted bacteria is most likely due to the accumulation of deleterious mutations by genetic drift, which also causes other moderately maladaptive consequences, including accumulation of deleterious amino acid substitutions and loss of adaptive codon biases [36], [42], [43].

Because the population structure of host-restricted bacteria only allows them to respond to natural selection when it's relatively strong (in relation to bacterial population size), the fact that these species are mostly responding to selection against spurious binding and not to selection for −10 site over-representation in regulatory regions allows us to gauge the relative strength of these two selective forces. The different response to selection suggests that the deleterious fitness effects of spurious binding are much larger than the potential benefits of finely tuned regulation of gene expression via over-representation of −10 sites, at least in host-associated bacteria. By an analogous reasoning, the low numbers of tRNA genes and the absence of adaptive codon biases in these species suggest that avoidance of RNAP spurious binding is a stronger constraint than optimization of speed and accuracy during translation.

### Conclusions

Unlike the highly localized selective pressures on amino acid sequence, selection to optimize gene expression can affect all portions of the genome, including sequences not directly involved in regulatory processes. The implications for genome sequence evolution are large, as this distributed selective process signifies that no sequence may be free of selective constraints, at least in the relatively small and unstructured genomes of bacteria [8]. In addition, our study suggests that different genomic traits can respond to specific selective pressures in an integrated manner. Understanding the coevolution among the different molecules, pathways, subsystems and processes of the cell will be of central importance as we enter a new era of synthetically designed genomes for biotechnology and medicine. In the same way that evolutionary studies of individual molecules have provided many insights into organismal adaptation, the analysis of whole genomes in a broad comparative framework has the potential to uncover such coevolutionary patterns.

## Methods

### Obtaining a list of −10 σ^70^ sites

Current information on all aspects of gene regulation in *E.coli* K12 is summarized in RegulonDB [25]. At the time we started these analyses, Regulon DB contained 584 experimentally detected σ^70^ promoters. The hexamers predicted to be the most likely −10 motifs in each of these 584 promoters were obtained using the program WCONSENSUS [44] and algorithms developed by Huerta and Collado-Vides [14]. This set of hexamers contained 185 different words that were used as −10 σ^70^ sites in all subsequent analyses (Table S1).

### Subdividing genomes into different regions

Completely sequenced bacterial genomes were downloaded from GenBank [45], and only chromosomal DNA was considered. Transcription units were defined within each genome according to a method which takes into account distributions of intergenic distances, and genomic DNA was then parsed into three categories: coding, regulatory, and nonfunctional [46]. Regulatory DNA was defined to be 250 bases upstream of the +1 start site of an operon or single transcription unit. 90% of the promoters in *E. coli* are known to occur within this range. Nonfunctional DNA was defined as sequence located between adjacent genes transcribed in convergent directions, which is not expected to contain promoter elements. Noncoding DNA located beyond 250 bp from +1 or between genes belonging to the same transcriptional unit was not included in the analysis, as the possibility that transcription might start within these sequences can not be excluded. Open reading frames over 1kb were included in the coding DNA category. For every genome, three sequence concatenations were produced by adjoining all the sequences in a DNA category. An “N” was inserted between concatenated sequences to avoid introducing artificial motifs at each concatenation point.

### Generating shuffled sequence

To obtain the neutral expectations for hexamer frequencies, we generated shuffled DNA with the same background biases as the original genomic sequences. To this aim we employed a strategy based on a limited eularian walk [47], which produced shuffled sequences that preserved the trinucleotide frequencies present in the original DNA. An algorithm written in Perl was produced to implement this approach and employed to independently shuffle the concatenations of regulatory, nonfunctional and coding sequences. Shuffling was restricted to 1Mb samples of the total concatenation when actual length exceeded this value (Tables S2, S3 and S4). We generated sets of 1000 shuffled sequences for each sequence category in every one of the genomes analyzed. As a procedural control, we also generated and shuffled a pseudo-random sequence totaling 1 Mb.

### Counting hexamers and establishing over- or under-representation

In order to establish whether −10 σ^70^ sites are under- or over-represented in each sequence category, we compared their observed counts to their expected occurrences given the base composition of each type of region. Neutral expectations were determined separately for each sequence category, by counting the occurrences of the −10 σ^70^ hexamers in sets of 1000 shuffled sequences. The number of occurrences of each of the 185 hexamers was independently counted within a given sequence using a sliding window approach, and the counts for each word were summed to obtain the total number of −10 σ^70^ sites. For every genome and sequence category, we obtained the mean and standard deviation of the total hexamer counts in the sets of 1000 shuffled sequences, and the observed hexamer counts were compared to these values. Over- and under-representation were considered significant when the observed counts lied beyond 2 Standard Deviations (SD) from the mean. One megabase of random sequence with no nucleotide biases was produced with Perl's random number generator and used as a control where the detected hexamer counts in the original sequence should closely approximate the frequencies obtained in the shuffled set.

### Phylogenetically-based correlation analyses

In order to detect possible covariation between different genomic properties, we performed correlation analyses employing comparative methodologies that take into account the phylogenetic relationships among the analyzed bacterial species. Species phylogeny was based on a Bayesian analysis of a set of 51 genes present in single copy in most bacterial genomes. This core of genes is best suited for deep phylogenetic reconstruction because it evolves under high and similar selective pressures across all organisms and maximally avoids gene loss, duplication, horizontal transfer and accelerations of evolutionary rate. Concatenated multiple sequence alignments were constructed using the MUSCLE 3.52 program [48], [49], and were used as input for the bayesian tree reconstruction program MrBayes [50]. MrBayes was run twice independently, using the JTT amino acid substitution matrix [51] and 4 gamma rate categories to model among-site rate variation. Each run included four Markov chains, three heated and one cold, starting from random trees and proceeding for 250,000 generations. The tree topology and branch lengths of the most likely tree obtained were used as input for CONTINUOUS, a computer program that implements the generalized least squares (GLS) model for across-species analysis of comparative data [31], [32].

## Supporting Information

Table S1 List of 185 unique −10 hexamers derived from 584 experimentally detected s70 E. coli promoters.
(0.04 MB DOC)

Table S2 Observed vs. expected counts of −10 motifs in regulatory regions. Total Sequence Length is the total number of base pairs analyzed for the corresponding type of region in each genome. NSD is the number of standard deviations that separate the observed motif count from the expected mean in that genome, NSD = (Obs-Mean)/SD.
(0.05 MB DOC)

Table S3 Observed vs. expected counts of −10 motifs in nonfunctional regions. Total Sequence Length is the total number of base pairs analyzed for the corresponding type of region in each genome. NSD is the number of standard deviations that separate the observed motif count from the expected mean in that genome, NSD = (Obs-Mean)/SD.
(0.07 MB DOC)

Table S4 Observed vs. expected counts of −10 motifs in coding regions. Total Sequence Length is the total number of base pairs analyzed for the corresponding type of region in each genome. NSD is the number of standard deviations that separate the observed motif count from the expected mean in that genome, NSD = (Obs-Mean)/SD.
(0.06 MB DOC)
