# Supplementary material for: Selection against Spurious Promoter Motifs Correlates with Translational Efficiency across Bacteria
Source: PLoS One. 2007 Aug 15;2(8):e745. doi: 10.1371/journal.pone.0000745 (PMC1939733; doi:10.1371/journal.pone.0000745)
Supplement: Table S1 — List of 185 unique −10 hexamers derived from 584 experimentally detected s70 E. coli promoters. (0.04 MB DOC) [file pone.0000745.s001.doc]

| **Table S1. List of 185 unique –10 hexamers derived from 584 experimentally detected σ^70^ *E. coli* promoters** | | | | |
| --- | --- | --- | --- | --- |
| CAAAAT | CATGAT | TAACAT | TACGAG | TATAAC |
| CAAACT | CATGGC | TAACCA | TACGAT | TATAAG |
| CAAATC | CATGTT | TAACCG | TACGCC | TATAAT |
| CAAATG | CATTAT | TAACCT | TACGGC | TATACG |
| CAAATT | CATTCG | TAACGG | TACGTC | TATACT |
| CAACCG | CATTCT | TAACGT | TACGTG | TATAGC |
| CAACCT | CATTGC | TAACTC | TACGTT | TATAGT |
| CAACGC | CATTTT | TAACTG | TACTAT | TATATT |
| CAAGAT | GAAAAT | TAAGAC | TACTCT | TATCAG |
| CAAGCC | GAAATC | TAAGAT | TACTGT | TATCAT |
| CAATAC | GAACGA | TAAGCT | TACTTG | TATCCG |
| CAATAT | GAATGG | TAAGGC | TACTTT | TATCCT |
| CAATGC | GACAAC | TAAGGG | TAGAAC | TATCGC |
| CAATGG | GACCGT | TAAGGT | TAGAAT | TATCGG |
| CAATGT | GACGAT | TAAGTC | TAGACT | TATCGT |
| CAATTC | GACGCC | TAAGTT | TAGAGT | TATCTA |
| CAATTG | GAGAAT | TAATAC | TAGATT | TATCTC |
| CACACT | GAGCGT | TAATAG | TAGCAG | TATCTT |
| CACCAT | GAGGGA | TAATAT | TAGCAT | TATGAA |
| CACCCT | GATGTG | TAATCA | TAGCCG | TATGAT |
| CACCGG | GATGTT | TAATCG | TAGCCT | TATGCG |
| CACTTT | GATTTT | TAATCT | TAGCTG | TATGCT |
| CAGACT | TAAAAA | TAATGC | TAGGAA | TATGGC |
| CAGATT | TAAAAC | TAATGT | TAGGAT | TATGGT |
| CAGCAT | TAAAAG | TAATTA | TAGGCG | TATGTC |
| CAGGAC | TAAAAT | TAATTC | TAGGCT | TATGTT |
| CAGGAT | TAAACC | TAATTG | TAGGGT | TATTAA |
| CAGGTT | TAAACT | TAATTT | TAGGTT | TATTAC |
| CAGTGT | TAAAGC | TACAAG | TAGTAC | TATTAT |
| CATAAT | TAAAGG | TACAAT | TAGTAT | TATTCC |
| CATACG | TAAAGT | TACACT | TAGTCG | TATTCT |
| CATACT | TAAATA | TACAGT | TAGTCT | TATTGT |
| CATAGT | TAAATC | TACATT | TAGTGG | TATTTA |
| CATCAG | TAAATG | TACCAT | TAGTGT | TATTTC |
| CATCAT | TAAATT | TACCCT | TAGTTG | TATTTG |
| CATCGT | TAACAA | TACCTG | TAGTTT | TATTTT |
| CATCTT | TAACAC | TACCTT | TATAAA | TGTCCT |
