# Supplementary material for: Selection against Spurious Promoter Motifs Correlates with Translational Efficiency across Bacteria
Source: PLoS One. 2007 Aug 15;2(8):e745. doi: 10.1371/journal.pone.0000745 (PMC1939733; doi:10.1371/journal.pone.0000745)
Supplement: Table S4 — Observed vs. expected counts of −10 motifs in coding regions. Total Sequence Length is the total number of base pairs analyzed for the corresponding type of region in each genome. NSD is the number of standard deviations that separate the observed motif count from the expected mean in that genome, NSD = (Obs-Mean)/SD. (0.06 MB DOC) [file pone.0000745.s004.doc]

| Table S4. Observed vs. Expected Counts of –10 Motifs in Coding Regions | | | | | |
| --- | --- | --- | --- | --- | --- |
|  |  |  |  |  |  |
| **Species** | **Total Sequence**  **Length (bp)** | **Observed**  **Counts** | **Expected Mean**  **Counts** | **SD** | **NSD** |
| A_aeolicus | 1000000 | 42400 | 43092 | 146.17 | -4.74 |
| A_tumefaciens | 1000000 | 26281 | 25753 | 125.16 | 4.22 |
| B_aphidicola | 567000 | 47977 | 25753 | 125.16 | -11.2 |
| B_burgdorferi | 1000000 | 77347 | 78798 | 175.74 | -8.26 |
| B_japonicum | 1000000 | 20544 | 78798 | 175.74 | 8.09 |
| B_melitensis | 1000000 | 28859 | 26958 | 128.25 | 14.82 |
| B_subtilis | 1000000 | 43794 | 43818 | 153.52 | -0.16 |
| C_acetobutylicum | 1000000 | 76439 | 77015 | 175.56 | -3.29 |
| C_crescentus | 1000000 | 17518 | 16811 | 108.88 | 6.49 |
| C_glutamicum | 1000000 | 29150 | 29017 | 136.06 | 0.97 |
| C_jejuni | 1000000 | 71188 | 70924 | 178.46 | 1.48 |
| C_muridarum | 973000 | 48547 | 50815 | 155.65 | -14.58 |
| C_perfringens | 1000000 | 77882 | 80014 | 181.51 | -11.75 |
| C_pneumoniae | 1000000 | 50713 | 51601 | 158.85 | -5.59 |
| E_coli_K12 | 1000000 | 36850 | 37386 | 138.12 | -3.88 |
| F_nucleatum | 1000000 | 79564 | 80834 | 177.48 | -7.16 |
| H_influenzae | 1000000 | 59338 | 59441 | 164.74 | -0.63 |
| H_pylori | 999000 | 52918 | 54399 | 160.21 | -9.25 |
| L_innocua | 1000000 | 57475 | 58484 | 159.82 | -6.32 |
| L_lactis | 1000000 | 56695 | 57448 | 158.21 | -4.76 |
| M_genitalium | 527000 | 34745 | 36640 | 121.79 | -15.56 |
| M_leprae | 1000000 | 25412 | 25330 | 128.37 | 0.63 |
| M_loti | 1000000 | 21599 | 20896 | 119.29 | 5.89 |
| M_pneumoniae | 722000 | 36051 | 37712 | 133.03 | -12.49 |
| N_meningitidis | 1000000 | 34993 | 34113 | 143.54 | 6.13 |
| Nostoc | 1000000 | 49840 | 51601 | 160.22 | -11 |
| P_aeruginosa | 1000000 | 17674 | 17194 | 112.03 | 4.28 |
| P_luminescens | 1000000 | 49826 | 50674 | 159.99 | -5.3 |
| P_multocida | 1000000 | 53820 | 54612 | 164.57 | -4.81 |
| R_conorii | 1000000 | 78918 | 80258 | 183.55 | -7.3 |
| R_solanacearum | 1000000 | 17919 | 18113 | 115.7 | -1.68 |
| S_aureus | 1000000 | 69910 | 71833 | 182.12 | -10.56 |
| S_meliloti | 1000000 | 21957 | 21239 | 119.11 | 6.03 |
| S_pneumoniae | 1000000 | 50423 | 50123 | 163.81 | 1.83 |
| S_typhimurium_LT2 | 1000000 | 37318 | 37129 | 132.2 | 1.43 |
| Synechocystis | 1000000 | 43597 | 43867 | 148.98 | -1.82 |
| T_maritima | 1000000 | 30679 | 30325 | 141.23 | 2.51 |
| T_pallidum | 999000 | 33363 | 33671 | 138.84 | -2.22 |
| U_urealyticum | 685000 | 59451 | 61861 | 147.93 | -16.29 |
| V_cholerae | 1000000 | 38745 | 39768 | 143.49 | -7.13 |
| W_glossinidia | 607000 | 56352 | 58760 | 139.96 | -17.21 |
| X_fastidiosa | 1000000 | 32708 | 33260 | 142.2 | -3.89 |

Total Sequence Length is the total number of base pairs analyzed for the corresponding type of region in each genome. NSD is the number of standard deviations that separate the observed motif count from the expected mean in that genome, NSD = (Obs-Mean)/SD.
